# Image-based closed-loop feedback for highly mono-dispersed microdroplet production

**DOI:** 10.1038/s41598-017-11254-5

**Published:** 2017-09-05

**Authors:** D. F. Crawford, C. A. Smith, G. Whyte

**Affiliations:** 1 Institute of Biochemistry, Biophysics and Bioengineering, Heriot-Watt University, Edinburgh, EH14 4AS UK; 2 Sphere Fluidics Limited, The Jonas-Webb Building, Babraham Research Campus, Babraham, Cambridge, CB22 3AT UK

## Abstract

Micron-scale droplets isolated by an immiscible liquid can provide miniaturised reaction vessels which can be manipulated in microfluidic networks, and has seen a rapid growth in development. In many experiments, the precise volume of these microdroplets is a critical parameter which can be influenced by many external factors. In this work, we demonstrate the combination of imaging-based feedback and pressure driven pumping to accurately control the size of microdroplets produced in a microfluidic device. The use of fast-response, pressure-driving pumps allows the microfluidic flow to be quickly and accurately changed, while directly measuring the droplet size allows the user to define the more meaningful parameters of droplet size and generation frequency rather than flow rates or pressures. The feedback loop enables the drift correction of pressure based pumps, and leads to a large increase in the mono-dispersity of the droplets produced over long periods. We also show how this can be extended to control multiple liquid flows, allowing the frequency of droplet formation or the average concentration of living cells per droplet to be controlled and kept constant.

## Introduction

The miniaturisation of experimental techniques and the growth of lab-on-a-chip systems have stimulated interest in microfluidics where networks of micron scale channels move and manipulate liquids. However, in general, the high surface to volume ratio of such channels leads to strong interactions with the walls, which can result in unintended dispersion and mixing. By encapsulating the sample in a second immiscible phase microdroplets can be produced within microfluidic networks. The immiscible carrier phase isolates each discrete droplet from those around it and eliminates direct contact with the walls. This provides a powerful platform capable of producing discrete reaction vessels for a wide variety of applications, including chemical^1, 2^ and biochemical reactions^3, 4^, library generation^5, 6^ and screening, nanoparticle fabrication^7, 8^. Additionally, individual particles, cells or molecules, can be encapsulated and isolated in microdroplets for studying them at the single level. This technique has been employed to capture single DNA molecules and perform polymerase chain reactions (PCR)^9–11^. Similarly single cell sequencing can be performed on individual cells held within microdroplets^5^.

The ability to successfully perform these experiments is dependent on the size and monodispersity of the microdroplets. This in turn depends on a wide variety of parameters, including flow rates, device geometry, viscosities, interfacial tensions, and so reliably creating droplets of a known size is often a trial-and-error process. Accurate control of droplet volume is critical in many applications of microdroplets where the changes in the volume give rise to errors in the estimation of the concentration of a product, or where encapsulating single objects, e.g. single molecules^12^ or cells^5^, is important.

Microdroplet size control is generally grouped into two categories, active and passive. Active methods for microdroplet size control use external manipulations to alter the size and have included embedding pneumatic valves^13^, electric fields^14^, piezoelectric elements^15^ and optical heating^16^ to control microdroplet production, but these techniques require advanced device production or complex external equipment.

Passive microdroplet control on the other hand uses continuously flowing immiscible liquids which create microdroplets. By changing the liquid properties (e.g. viscosity or interfacial tension), channel geometry or more simply the flow rate, the resulting droplet size can be altered over a wide range. The two main configurations for microdroplet production are the T-junction^17^ and the flow-focussing junction^18^. Both syringe^19^ and pressure pumps^20^ can be used to control the input liquids. However, the response time of syringe pumps may be seconds or even in some cases minutes^21^ which limits the ability to quickly change the droplet size. Pressure-based pumping can quickly alter the microdroplet generation sizes in a fraction of a second, however the pressure required at each inlet depends on backpressure, which in turn depends on the device design, the diameter and length of tubing and liquid properties, resulting in strong dependence on experimental conditions and possible drift throughout the experiment.

To counter these problems, a pressure-pump feedback system has been developed which uses images of the droplets being formed to feedback and control the droplet size. By using pressure-pumps, rapid changes can be actuated and by measuring the droplet size directly, any drift can be compensated, providing ultra-monodispersed microdroplets created in an intuitive and easy-to-use fashion. Previous feedback systems^22^ have used syringe-pumps resulting in a long response time (100’s of seconds) and high polydispersity^23^. By implementing two pressure pumps, changes in pressure, and therefore changes in flow rates, will have a faster response making a closed loop feedback system able to perform at higher throughputs.

**Figure 1:**
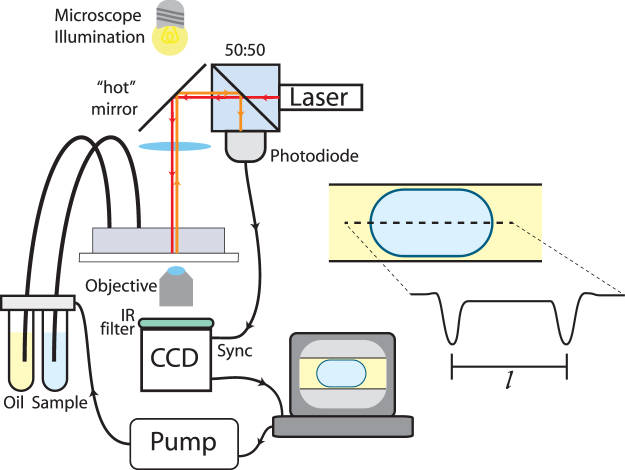
Schematic diagram showing the setup used for image-based feedback. Two immiscible phases (fluorous oil and water) are pumped into a microdevice featuring a flow-focussing section, by pressurised air controlled by bespoke feedback software. Image acquisition is synchronised to the presence of a droplet using the backscatter signal from a IR laser. The droplet length can be measured using simple image processing, the volume can be calculated and this information fed back to the control of the pump.

The system was set up as shown in Fig. 1, where a PDMS microfluidic device with a flow-focusing junction was mounted on a microscope equipped with a high-speed camera synchronised to droplet formation using an infra-red laser and back-scatter detector. By synchronising the camera to the presence of droplets, droplet size measurements can be made simply by measuring the droplet in the middle of the camera image. The droplet volume is calculated from the length of the droplet and this information is used in the feedback loop to control the applied pressures on the aqueous and oil inlets.

## Results and Discussion

**Figure 2:**
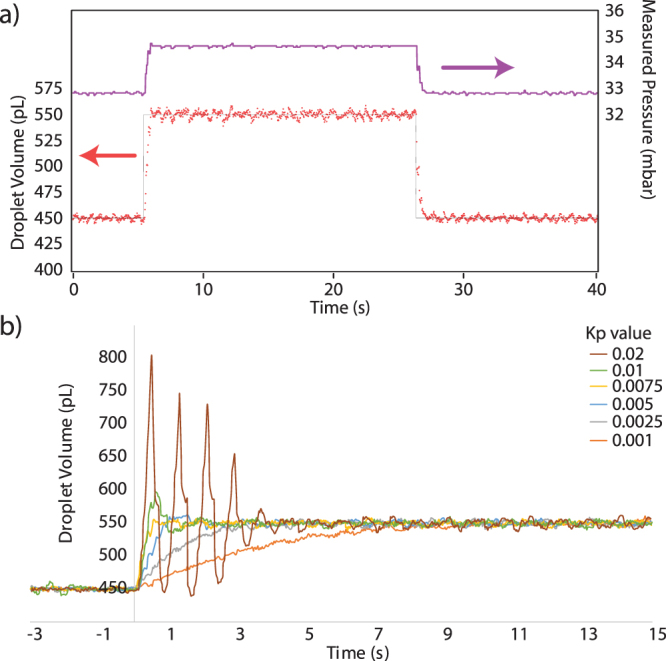
(**a**) Response of the droplet formation to a change in wanted droplet volume. The volume of each droplet created (red dots) quickly follows the wanted droplet profile (black line) as the pressure at the aqueous inlet is changed (purple line). (**b**) Optimisation of the dominant feedback parameter Kp, for a step change in wanted droplet volume from 450 to 550 pL, showing the slow responses at low values and oscillation at large values.

As can be seen in Fig. 2, the use of a feedback loop allows the rapid and well defined change in the generated droplet volume by rapidly altering the applied pressure. Figure 1a shows an example where the target volume was changed between 450 pL and 550 pL. The software rapidly changes the applied pressure and droplets are formed with the new volume within 500 milliseconds. At these formation rates, this results in 16 droplets having a volume in between the two wanted values.

As with all feedback based systems, the control parameters of the feedback loop are critical to a fast response system. The parameters need to be chosen to provide the optimum conditions between a slow response and overshooting and oscillation; Fig. 1b shows the response to a change in the wanted volume from 450 to 550 pL for different values of *K*_*p*_. As expected, low *K*_*p*_ values (0.001, 0.0025) result in a slow response to the desired change, while very high values (0.01, 0.02) result in oscillations around the target value. Changes in *K*_*d*_ and *K*_*i*_ had smaller effects on the response and for the rest of the work, the values were kept at *K*_*p*_ = 0.005, *K*_*d*_ = 0 and *K*_*i*_ = 0.

**Figure 3:**
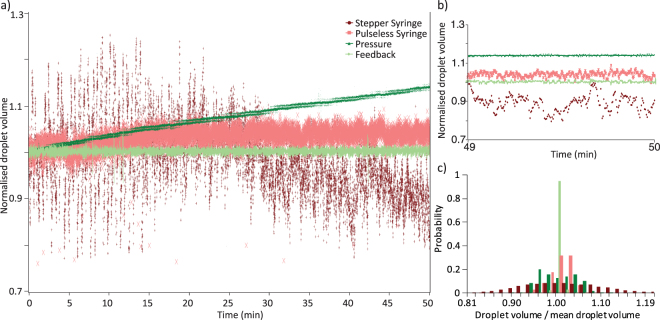
(**a**) Normalised droplet volumes over a 50 minute timescale created using constant flow rate, stepper motor driven syringe pumps (dark red), pulseless syringe pumps (light red), constant pressure pumps (dark green) and image-based feedback driving a pressure pump, producing droplets at 50 Hz (light green). The improvement in monodispersity of the pressure based systems and the drift cancelling effect of the feedback can be clearly seen. (**b**) Detailed view of the changes at short timescales, showing the oscillations due to the syringe pump and drift due to the constant pressure pump. c) Normalised histograms of the normalised droplet volume for each of the methods shown.

To investigate how this system can improve the monodispersity of microdroplet formation, the droplet volume was measured using four different pumping systems, a stepper motor driven syringe pump, a pulseless syringe pump, a constant pressure air over liquid pump, and the developed image-based feedback system. For each system a minimum of 15,000 droplets were measured. Figure 3 shows that the distribution of microdroplet sizes created using the stepper motor driven syringe pump is relatively broad with periodic oscillations. This behaviour can be understood in terms of the discrete movements of the stepper motor which drives the syringe^24, 25^. Significantly narrower distributions can be achieved using pulseless syringe pumps which do not show the same oscillations, however when compared to pressure driven pumps which apply a constant pressure, the distribution is still very broad. Although the applied pressure remains constant through the experiment, the flow rates in the device may change due to channel fouling, leaking or the height of the liquid in the outlet changing, which can lead to long-term drift in the droplet volume (see Fig. 2a). This can be countered by using a flow meter placed in the fluid path, with a closed feedback loop, however the droplet size produced in using a constant flow rate system can also change due to temperature changes, fouling or surface wetting effects. Additionally, the flow meters are often limited in the range of liquids which can be used, and can be easily blocked by objects in the liquid such as microparticles or cells, thus limiting their applicability.

Imaging the droplets provides a more accurate metric of the droplet size than the input flow rates, and by feeding back this information, it is possible to improve the long-term monodispersity of the droplet production, as shown by the distribution data shown in Table 1, without placing anything in the fluid path. The standard deviation in the volume of droplets created using image based feedback is significantly improved over the other methods tested and even when compared to using the pressure pumps the standard deviation reduces from 3.78% to only 0.32% for 300 pL droplets created over a 50 minute period. This value is close to the resolution limit of our system, which was measured to be the equivalent of 0.19%.

**Table 1:** measurements of the distributions in droplet volume measured over a 45 minute period showing the improvement in monodispersity when using acive feedback to control droplet production.

	Coefficient of Variation	Fraction of droplets within percentage of mean volume		
		*5%*	*1%*	*0*.*50%*
Stepper motor syringe	10.98%	41%	9%	*4%*
Pulseless syringe	5.42%	97%	30%	*17%*
Pressure	3.78%	82%	13%	*6%*
Feedback	0.32%	100%	99.70%	*88%*
Frequency feedback	0.40%	100%	98.60%	*80%*

**Figure 4:**
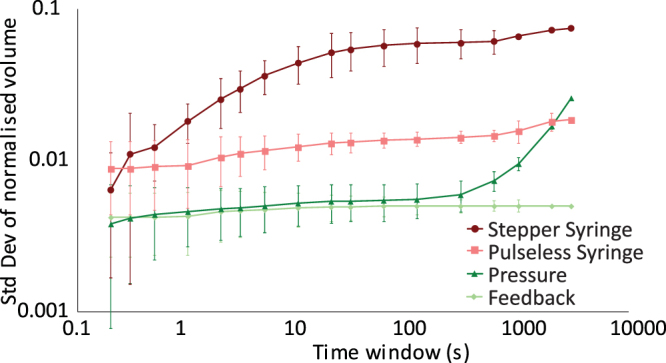
Changes in the distribution of droplet sizes taken for different time windows for the syringe pumps (red and pink), constant pressure pumps (dark green) and image-based feedback controlling pressure pumps (light green). At short timescales the two pressure based systems perform identically, however over long time periods, the drift of the constant pressure system becomes apparent while the image-based feedback remains constant.

**Figure 5:**
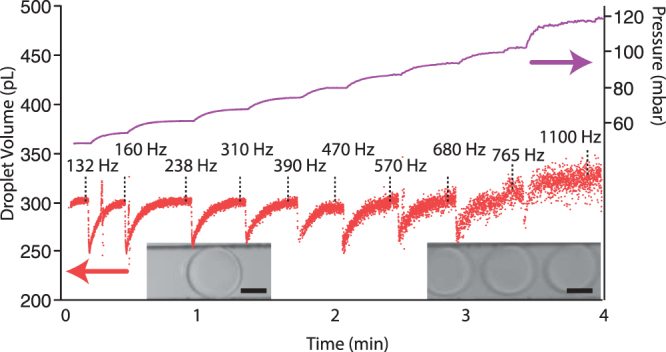
Droplet feedback maintaining droplet volume (red dots) at high droplet formation rates. The oil pressure was increased stepwise and the feedback system was allowed to optimise the aqueous pressure (purple line) to form 300 pL droplets. At higher pressures, the increase in the error in the droplet volume is in part due to motion blue from the fast moving droplets. Insets: images of the droplets at low flow rates (left) and high flow rates (right) showing the increased motion blur. Scale bars are 50 µm.

By measuring the standard deviation of the distribution of microdroplet sizes within a given time window, the timescales over which the system is changing can be investigated (Fig. 4). As expected, the syringe pumps show a larger distribution even at short timescales, while the two pressure pump systems have very similar responses over short timescales, but due to compensating the inherent drifting of the flow rates, the feedback results show consistent monodispersity across the range of times measured. To test the capabilities at higher droplet production rates, the oil pressure was increased in a series of steps and the feedback algorithm modified the aqueous pressure to maintain the wanted droplet volume of 300 pL. Figure 5 shows that at low pressures the algorithm can maintain the wanted droplet volume at high production rates. Due to the time taken to transfer the images, process them and save the data, the processing loop is limited to ~250 Hz, thus the size of every droplet cannot be measured at droplet formation rates above this. However, an assumption that the imaging captures a representative sample of droplets can be made. Fast camera imaging of droplet formation at high flow rates confirms the assumption that the variation of droplet volumes within 0.1 s is smaller than the variation seen at the second timescale. At very high pressures (>100 mbar per channel), the linear droplet speed through the channel induces motion blur of the image, resulting in higher uncertainty in the droplet volume. Shorter exposure times (the camera used in this study was limited to 40 µs) or stroboscopic illumination would reduce this effect.

The use of line cameras to image the droplet would simplify the processing and increase the possible frame rate, allowing faster responses and quality assurance by measuring the size of every droplet, even at high formation rates. The relatively simple processing required to implement this system could also be performed by a microprocessor and integrated directly with the pressure pump to provide a straightforward system for generating droplets of a given size. Using the faster frame rate, droplet formation remained stable for frequencies up to 1KHz.

**Figure 6:**
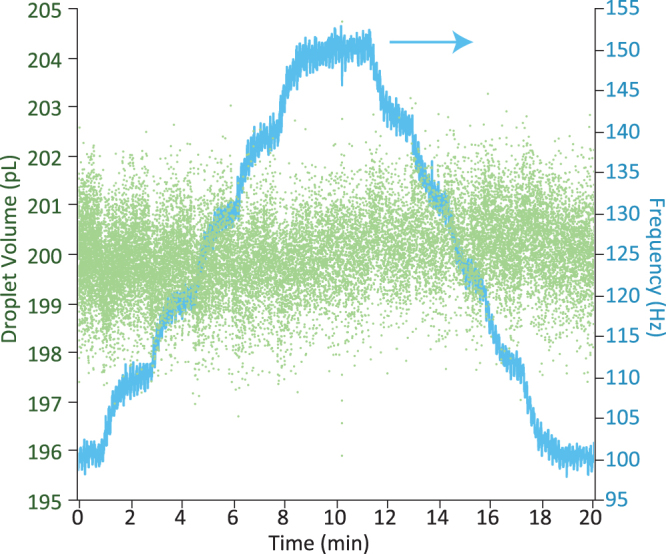
Response of the frequency (blue) and droplet volume (green) due to the frequency feedback system which controls both the aqueous and oil pressures to allow feedback control of both volume and frequency. The required frequency was increased in increments of 10 Hz from 100 Hz to 150 Hz and then back down to 100 Hz with only slight effects on the resulting droplet volume.

The system is not limited to a single feedback pressure, to demonstrate this, a secondary feedback loop was added to alter the oil pressure in order to control the frequency of droplet generation. Such a system gives the user control of both the volume and frequency of droplet production. The droplet frequency was measured from the back-scatter signal which was monitored by a microprocessor which triggered a counter on each droplet passing. The frequency can be calculated by counting the number of triggered voltages per second. The software then controllably alters pressures accordingly until the required droplet frequency is obtained. By tuning the feedback parameters, it is possible to prioritise the different measurements. To maintain the high monodispersity, it is important that the feedback loop of the volume acts faster than the frequency, allowing the volume to stay constant even while changing the oil pressure. The optimum *K*_*p*_ value for the frequency was found to be 0.0025, smaller than the *K*_*p*_ for the volume, resulting in the time taken for the frequency to stabilise after a change to be about 30 seconds, as shown in Fig. 6. The uncertainty in the volume remains at about 0.4% and so is unaffected by the fluctuations of the pressure pumps caused by the frequency feedback loop.

**Figure 7:**
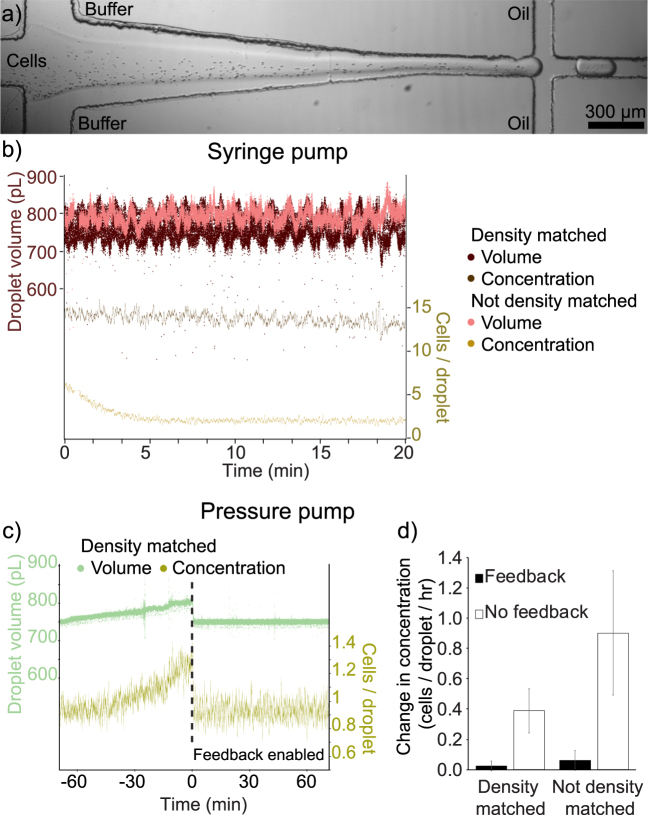
Extension of the feedback system to stabilise average cell encapsulation concentrations in microdroplets. (**a**) Micrograph of the cell encapsulation device, consisting of an aqueous inlet for cell suspension samples which is diluted and flow focused by a dilution buffer, followed by flow focussing with an immiscible oil to form microdroplets. (**b**) Graph showing the variation in droplet volume (red) and average red blood cell count per droplet (yellow) when using syringe pumps. Cells placed in a syringe without density matching quickly settle and the concentration of cells encapsulated quickly falls (yellow curve). By density matching the sample to the cells, there is a slow drift in encapsulation concentration. (**c**) Graph showing the change in volume (green) and cell count per droplet (yellow) for pressure based pumps without feedback (time < 0) and with feedback (time > 0). Drift in the cell count is also present when using pressure pumps, as can be seen before feedback is turned on at time = 0, however this can be compensated for by feedback to alter the dilution factor. (**d**) Extracted rate of change of the cell count per droplet when using pressure pumps with and without feedback for density matched and non density matched red blood cells. The plots show the mean gradient of linear fits to the cell count per droplet over a thirty minute period, error bars are the standard deviation (N = 3). Scale bar is 300 µm.

To further demonstrate the potential for image-based closed loop feedback, the feedback loop was modified to control two aqueous inlets in order to stabilise the concentration of cells encapsulated into microdroplets. The device consisted of two flow focus junctions, one to dilute the cell sample with additional buffer, and the other to produce microdroplets as described above (Fig. 7a). When cells are placed into a vial or syringe at the inlet of a microfluidic device, they will sediment due to the difference in density between the surrounding medium and the cells. This results in a changing cell concentration being introduced into the device. The nature of this change depends on the experimental setup, but is generally a decrease in concentration when using syringes and an increase when using vials. This effect can be reduced by density matching the surrounding media with supplements such as Percoll^4^ and OptiPrep^26, 27^, however the inhomogeneous density of a cell sample and small errors in preparations means there will always be a drift in cell concentration (Fig. 7b). Image-based feedback can be used to compensate for any drift by dynamically altering the dilution on-chip. To do this, the oil pressure was kept constant while the feedback loop used the droplet volume to determine the total pressure for both aqueous inlets, and the cell concentration was used to determine the ratio of the two aqueous inlet pressures. By controlling both these parameters, the drift in droplet volume as well as cell concentration can be compensated for as shown in Fig. 7c and d. Although the droplet volume drifts over time (~6% per hour), this cannot fully account for the change in cell concentration (~40% per hour) and so two feedback conditions are required to maintain monodispersed volumes and cell concentrations.

## Experimental

### Microdevice Fabrication

PDMS devices were fabricated using SU-8 photolithography techniques as described elsewhere^28^. Briefly, SU-8 masters with 50 µm height are produced by spin coating Si wafers with SU-8 2025 (MicroChem Inc) and exposed to UV light to initiate crosslinking. Remaining SU-8 is removed using developer solvent (MicroChem Inc). Liquid PDMS (Sylgard 184) mixed in a 10: 1 elastomer: curing agent is poured onto the master. The PDMS is baked in a 60 °C oven for 3 hrs before being cut out and access holes punched using a biopsy puncher. The device and corresponding glass slide, to be used for the base of the channel, are treated with an air plasma (Diener) and brought into contact to seal. The surface of the device is treated with PicoGlide (Sphere Fluidics) by flushing the solution into the channels and then clearing with compressed air, to render the surfaces hydrophobic.

### Microscope setup

The droplet formation is observed using a camera (ProSilica GE680, AVT for Figs 2–6 or 340 M, Thorlabs for Fig. 7) mounted on an inverted microscope (AE31, Motic) and imaged using either a 10× objective (Figs 2–6) or a 4× objective (Fig. 7). In order to synchronise the camera to droplet formation, an 850 nm diode laser is coupled into the illumination path above the condenser lens. The coupling is achieved using a dichroic short pass filter “hot mirror” (M254H45, Thorlabs) which allows the visible microscope illumination to pass into the condenser as normal, while reflecting the infra-red light of the laser which is directed perpendicularly to the illumination axis. The filter is mounted on a kinematic stage (B4C, Thorlabs) to allow the position of the laser spot to be moved within the field of view. The laser was aligned to the middle of the field of view with a spot size in the focal plane of ~30 microns. The backscattered light collected by the condenser lens is reflected from the dichroic filter and directed towards a photodiode (DET36A, Thorlabs) using a 50:50 non-polarising beam splitter. The signal from the photodiode is fed into an analogue input of an Arduino microprocessor (Geniuno, Arduino) which in turn generates a trigger pulse signal for the camera when it detects a change in the backscattered light due to the presence of a droplet. The trigger signal is also fed into the counter input of a DAQ card (USB-6221, National Instruments) to track the droplet frequency.

### Droplet Volume Estimation

The length of the droplet, parallel to the channel, across its centre is measured using a line intensity profile. The NI-IMAQ peak detection tool is implemented to locate the edges of each droplet allowing length, L to be calculated. This length is used to estimate the droplet volume, ${V}_{measured}$, using the follow approximation^29^:

$${V}_{measured}=(HW-(4-\pi ){(\frac{2}{H}+\frac{2}{W})}^{-2})(L-\frac{W}{3})$$

Where H is the channel height and W is the channel width. For cases where the measured droplet length is smaller than either channel dimension, the volume was calculated as a sphere of radius *L/2*.

### Feedback system

The droplet volume measured, as described above, is averaged over the last 50 droplets to give $\overline{{V}_{measured}}$ and compared to the wanted value, ${V}_{wanted}$. The error, ${\sigma }_{V}=\overline{{V}_{measured}}-{V}_{wanted}$, *i*s used to modulate the pressure pumps.

Droplet volume measurements are integrated over a timescale of 0.1 s to reduce the effects of outliers. The change in pressure, *Δ*, is governed by the feedback equation^30^:

$${\rm{\Delta }}={K}_{p}^{V}{\sigma }_{V}+{K}_{i}^{V}\int {\sigma }_{V}dt+{K}_{d}^{V}\frac{d}{dt}{\sigma }_{V}$$

Where ${K}_{p}^{V}$, ${K}_{i}^{V}$ and ${K}_{d}^{V}\,$are the proportional, integral and derivative constants respectively, which describe the behaviour of the feedback. The value of,$\,{K}_{p}^{V}$, reacts proportionally to any change in the error reducing the rise time of any steady state errors. ${K}_{i}^{V}\,$reacts to and reduces long term, steady state errors, while ${K}_{d}^{V}$ is based on the rate of change of the error and reduces overshoots in the response of the feedback^31^.

In this work ${K}_{p}^{V}$ was found to be the dominant term and optimised as described in the main text. Therefore an error, *σ*, would result in a pressure change of ${\rm{\Delta }}={K}_{p}^{V}{\sigma }_{V}$ and so ${P}_{n+1}={P}_{n}+{\rm{\Delta }}$ where ${P}_{n}$ is the initial pressure provided by the pressure pump and ${P}_{n+1}$ is the new updated pressure. The response rate from the OB1 is 9 ms with a settling time of 40 ms. This allows the pressure change to be carried out in 0.05 s.

The cell concentration feedback loop used two parameters, ${P}_{n}^{T}$, representing the total aqueous pressure, and ${\alpha }_{n}$, the ratio of the cells to buffer pressure, where $n$ is the iteration number. Cell concentrations, $Con{c}_{measured}$ were determined in real-time from image analysis of camera image by background subtraction, thresholding and binary particle analysis using Vision Development Module (National Instruments). The feedback loop calculated the new errors and new values as:

$$\begin{array}{rcl}{\sigma }_{V} & = & \overline{{V}_{measured}}-{V}_{wanted}\\ {\sigma }_{C} & = & \overline{Con{c}_{measured}}-Con{c}_{wanted}\\ {P}_{n+1}^{T} & = & {P}_{n}^{T}+{K}_{P}^{V}{\sigma }_{V}\\ {\alpha }_{n+1} & = & {\alpha }_{n}+{K}_{p}^{\alpha }{\sigma }_{C}\\ {P}_{cells} & = & {P}_{n+1}^{T}.{\alpha }_{n+1}\\ {P}_{buffer} & = & {P}_{n+1}^{T}.(1-{\alpha }_{n+1})\end{array}$$

where ${\sigma }_{V},\,{\sigma }_{C}$ are the errors in the volume and concentration, ${K}_{p}^{V},\,{K}_{p}^{C}$ are the proportional feedback constants for the volume and concentration respectively and ${P}_{cells},\,{P}_{buffer}$ are the pressures applied to the cells inlet and buffer inlet respectively.

## Experimental Procedure

### Water-in oil droplet generation

Solutions of fluorous oil (2% PicoSurf, Sphere Fluidics in Novec 7500) and deionised water are delivered to the devices using silicon tubing with an OD (1 mm) and connected to either syringes with 22 gauge needles, or pressurised reservoirs (Elveflow). The needles are connected to a 1 mL syringe mounted on the corresponding stepper-based syringe pump (KD Scientific) or a pulseless syringe pump (Cetoni neMESYS), while the reservoirs are attached to a pressure pump (OB1 Elveflow). The outlet of the device is connected by tubing to a vial (2 mL Eppendorf) for collection. The laser is aligned to the middle of the channel several hundred microns from the flow-focussing junction and the camera’s region of interest is adjusted to the same position, to ensure only fully formed droplets are imaged. The threshold for triggering the camera is adjusted to ensure a single trigger pulse is sent for each droplet. Initial flow rates or pressures are chosen based on previous experience and allowed to equilibrate before starting the measurements. Images are acquired using the Vision Acquisition Software module of LabView (National Instruments) and processed in real time using the Vision Development Module of LabView. Control of the OB1 pressure pumps was achieved using the drivers supplied by the manufacturer which allowed direct control of the wanted pressure within LabView.

### Cell encapsulation

Red blood cell samples were prepared by dilution 10 µL of whole blood obtained by finger prick from healthy volunteers in accordance with Heriot-Watt University ethical guidelines with informed consent, into 1 mL of buffer solution consisting of either 28% OptiPrep solution in PBS (“Density Matched” buffer) or 10% OptiPrep in PBS (“Non Density Matched” buffer). The 28% OptiPrep solution was produced to have a density of 1.09 g/mL to match that of RBCs, 1.09–1.10 g/mL^32^. Samples were then loaded into either a syringe or vial and the procedure followed as described above.

## Conclusions

By using an imaging system to directly measure the microdroplet volume and using this to feedback control over the input pumps, it is possible to improve the monodispersity in volume of the microdroplets formed over long periods. The combination of closed-loop feedback and fast-response, pulseless pressure-driven pumping allows highly mono-dispersed microdroplet samples to be created without prior knowledge of the fluidic properties of the liquids or system. Over a short timescale (seconds) the standard deviation of the pressure pump system both with and without feedback is similar, showing that the monodispersity of the droplets does not suffer in these small time periods. Over long periods the feedback system corrects any drift that may occur and the pressure pump system produces droplets with average volumes less noisy than that of syringe pump based systems. Additional feedback parameters can be added to further extend the control over droplet generation as shown by controlling the frequency of microdroplet generation or the concentration of living cells encapsulated, to provide an easy to use and intuitive microdroplet generation system.

### Data Availability Statement

The datasets generated during the current study and the software developed are available from the corresponding author on reasonable request.

## Electronic supplementary material

Supplementary Information

## References

[1] Huebner A (2008). Microdroplets: a sea of applications?. Lab Chip.

[2] Theberge AB (2009). Suzuki-Miyaura coupling reactions in aqueous microdroplets with catalytically active fluorous interfaces. Chem. Commun. (Camb)..

[3] Courtois F (2008). An integrated device for monitoring time-dependent *in vitro* expression from single genes in picolitre droplets. Chembiochem A Eur. J. Chem. Biol..

[4] Huebner A (2008). Development of quantitative cell-based enzyme assays in microdroplets. Anal. Chem..

[5] Macosko EZ (2015). Highly Parallel Genome-wide Expression Profiling of Individual Cells Using Nanoliter Droplets. Cell.

[6] Theberge AB, Whyte G, Huck WTS (2010). Generation of picoliter droplets with defined contents and concentration gradients from the separation of chemical mixtures. Anal. Chem..

[7] Duraiswamy, S. & Khan, S. A. Plasmonic nanoshell synthesis in microfluidic composite foams. *Nano Lett*., doi:10.1021/nl102478q (2010). 10.1021/nl102478q 20731386

[8] Salmon, A. R. *et al*. Monitoring Early-Stage Nanoparticle Assembly in Microdroplets by Optical Spectroscopy and SERS. *Small* 1–9 doi:10.1002/smll.201503513 (2016). 10.1002/smll.201503513 26865562

[9] Zhang Y, Jiang HR (2016). A review on continuous-flow microfluidic PCR in droplets: Advances, challenges and future. Anal. Chim. Acta.

[10] Beer NR (2007). On-chip, real-time, single-copy polymerase chain reaction in picoliter droplets. Anal. Chem..

[11] Zhu Z (2012). Single-molecule emulsion PCR in microfluidic droplets. Anal. Bioanal. Chem..

[12] Hindson BJ (2011). High-throughput droplet digital PCR system for absolute quantitation of DNA copy number. Anal. Chem..

[13] Abate AR, Weitz DA (2008). Single-layer membrane valves for elastomeric microfluidic devices. Appl. Phys. Lett..

[14] Link DR (2006). Electric control of droplets in microfluidic devices. Angew. Chemie - Int. Ed..

[15] Bransky A, Korin N, Khoury M, Levenberg S (2009). A microfluidic droplet generator based on a piezoelectric actuator. Lab Chip.

[16] Baroud CN, de Saint Vincent MR, Delville J-P (2007). An optical toolbox for total control of droplet microfluidics. Lab Chip.

[17] Guillot P, Colin A (2005). Stability of parallel flows in a microchannel after a T junction. Phys. Rev. E.

[18] Anna SL, Bontoux N, Stone HA (2003). Formation of dispersions using ‘flow focusing’ in microchannels. Appl. Phys. Lett..

[19] Nisisako T, Torii T, Higuchi T (2002). Droplet formation in a microchannel network. Lab Chip.

[20] Faustini M (2013). Microfluidic approach toward continuous and ultrafast synthesis of metal-organic framework crystals and hetero structures in confined microdroplets. J. Am. Chem. Soc..

[21] Abate AR, Romanowsky MB, Agresti JJ, Weitz DA (2009). Valve-based flow focusing for drop formation. Appl. Phys. Lett..

[22] Miller E, Rotea M, Rothstein JP (2010). Microfluidic device incorporating closed loop feedback control for uniform and tunable production of micro-droplets. Lab Chip.

[23] Zeng, W., Li, S. & Wang, Z. Characterization of syringe-pump-driven versus pressure-driven microfluidic flows. in *2015 International Conference on Fluid Power and Mechatronics* (*FPM*) 711–715 doi:10.1109/FPM.2015.7337207 (IEEE, 2015).

[24] Li Z, Mak SY, Sauret A, Shum HC (2014). Syringe-pump-induced fluctuation in all-aqueous microfluidic system implications for flow rate accuracy. Lab Chip.

[25] Zeng W, Jacobi I, Li S, Stone HA (2015). Variation in polydispersity in pump- and pressure-driven micro-droplet generators. J. Micromechanics Microengineering.

[26] Ford T, Graham J, Rickwood D (1994). Iodixanol: A Nonionic Iso-osmotic Centrifugation Medium for the Formation of Self-Generated Gradients. Anal. Biochem..

[27] Mazutis L (2013). Single-cell analysis and sorting using droplet-based microfluidics. Nat. Protoc..

[28] Kolb T, Albert S, Haug M, Whyte G (2014). Dynamically reconfigurable fibre optical spanner. Lab Chip.

[29] Musterd M, van Steijn V, Kleijn CR, Kreutzer MT (2015). Calculating the volume of elongated bubbles and droplets in microchannels from a top view image. RSC Adv..

[30] Aström, K. J. & Murray, R. M. *Feedback systems: an introduction for scientists and engineers*. (Princeton university press, 2010).

[31] Arulmozhiyal, R. & Kandiban, R. Design of fuzzy PID controller for brushless DC motor. in *Computer Communication and Informatics* (*ICCCI*), *2012 International Conference on* 1–7 (IEEE, 2012).

[32] Carter, A. M. Platelet Proteomics Principles, Analysis, and Applications. 73 (2012).

