# Supplementary material for: Image-based closed-loop feedback for highly mono-dispersed microdroplet production
Source: Sci Rep. 2017 Sep 5;7:10545. doi: 10.1038/s41598-017-11254-5 (PMC5585215; doi:10.1038/s41598-017-11254-5)
Supplement: Supplementary file 1 — Supplementary Information [file 41598_2017_11254_MOESM1_ESM.pdf]

Image-based closed-loop feedback for highly mono-dispersed microdroplet production

D. F. Crawford, C. A. Smith and G. Whyte

Supporting data

To confirm that our feedback system provided consistent results, repeats of droplet creation were performed. Different PDMS devices with identical designs were used with new tubing, new oil and new water with the same software. As can be seen in Figure S1, without the feedback there is a constant drift of variable gradient (orange and red data) while with the feedback, the droplet volume variably remains around 1% of the mean. There is no drift over time as observed when no feedback loop in applied (blue data). Droplet volume was again measured using the pressure pumps with no feedback applied (orange and red data). As seen in Figure S1 the mean volume of the droplets increases over time. The rate at which this drift occurs was measured by linearly fitting the data. Gradients of 0.0201 s^{-1} (red data), 0.0079 s^{-1} (light orange data) and 0.0084 s^{-1} (dark orange data) were measured. Although the drifting effect occurs in all cases the rate at which this occurs is not. Without a consistent drift rate, a linear fix cannot be applied to compensate for the increase in droplet size. The feedback system must be applied to repeatedly adjust the pressure pumps to accommodate for this drift.

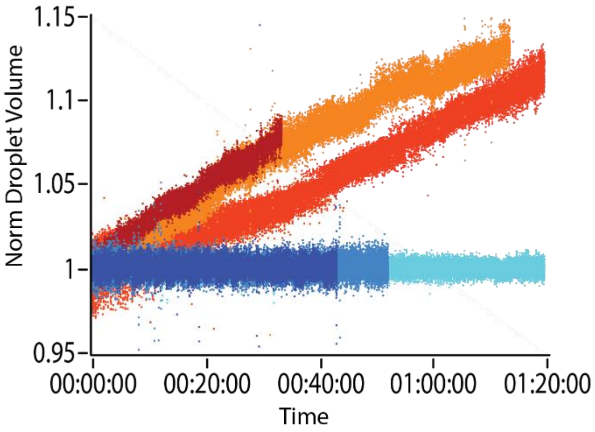

Figure S1 Repeats of droplet creation using pressure pumps both with and without feedback. Droplets were created on different days using different devices of identical design. The repeats without feedback (red and orange) drifted at different rates, while the repeats using the feedback system continued to stay around the required droplet volume.

To confirm that the droplet volume being measured within the device using the imaging feedback system was correct, droplets were created at various volumes and measured. The droplets were collected from the outlet of the device and placed onto a glass slide. Images were taken using a camera (ProSilica GE680) and measured using an image processing program (ImageJ). The diameter of the droplet was measured in pixels, using ImageJ, converted into the physical size and finally the volume for each droplet was calculated assuming the droplet is a sphere. 33 droplets for each volume were measured for the five selected input droplet volumes. The measured droplet size was plotted against the expected droplet size in figure S2. The droplet size was found to be consistent with the expected size with a standard deviation of less than 1% for each droplet size measured. The gradient was measured to be 0.9901 and if the trend line is clamped to the origin it gives a gradient of 0.9985. Figure S3 shows the difference between the expected and measured droplet volumes against the expected droplet volume. This contains the error bars seen in Figure S2, calculated using the standard deviation of

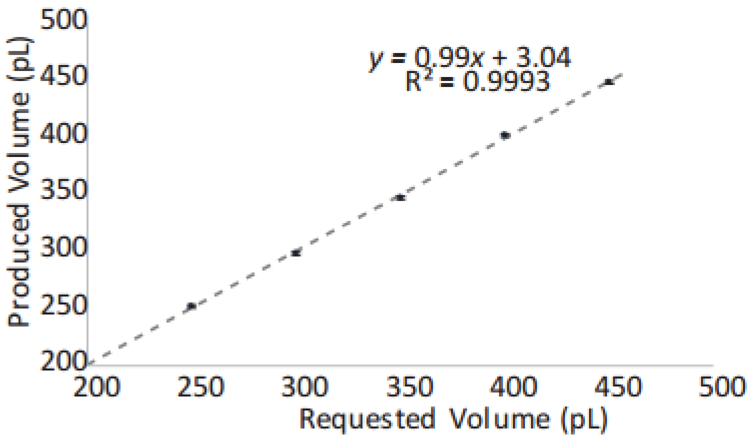

Figure S2. The droplet size produced by the pressure pump with feedback system was confirmed by comparing the input droplet volume controlled by the image feedback system and by removing the droplets from the device and measuring them on a glass slide. The droplet size measured was consistent with that of the input volume. Error bars are present and were calculated using the standard deviation of the measured droplet sizes.

measured droplet volumes. A negligible gradient of -0.0099 and sporadic position of points both above and below the x-axis shows there is no systematic error in the volume calculation.

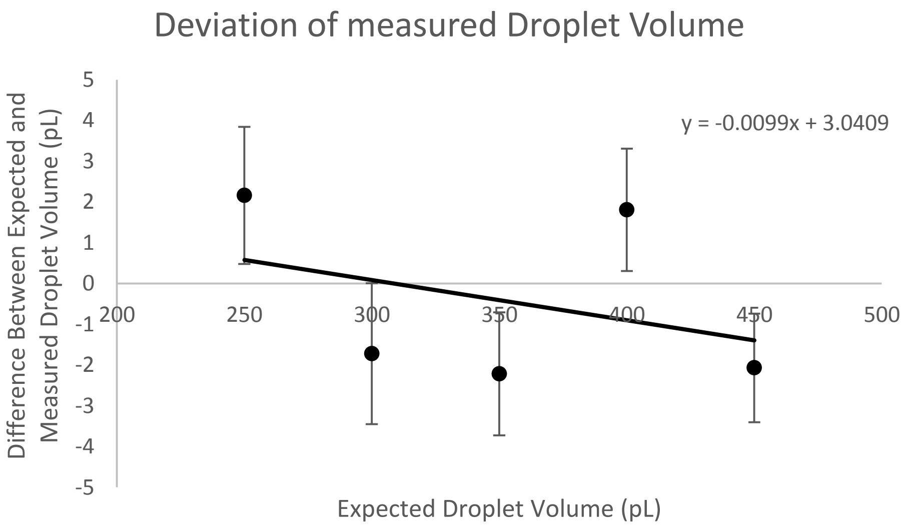

Figure S3. The deviation from the required volume of the measured droplet volume is shown. The error bars represent the standard deviation of the deviation of each measured droplet.

As the system was pushed to higher and higher frequencies motion blur became an issue with multiple microdroplets merging into one image and therefore giving an inaccurate droplet length. A strobe light source was able to solve this problem. Uniform droplet formation was stable for frequencies up to 1 kHz but at these high droplet formation frequencies the frequency was found to drift with time (blue data points in figure S4). This was corrected using the frequency feedback system and the frequency remained more stable (red data points in figure S4). In both cases there was no change in droplet volume between the two experiments, due to the volume feedback loop still being present. The standard deviation of the 200 pL droplets created, both with and without frequency feedback, at 1 kHz was 2.4 pL (1.2%). This is higher than the standard deviation presented in Table 1 and is due to the reduced light levels when using the 1 µs strobe illumination and the resulting lower signal to noise ratio. At droplet formation frequencies between 50 Hz and 1000 Hz, the standard deviation was consistent, showing the droplet volume accuracy was not reduced at higher frequencies.

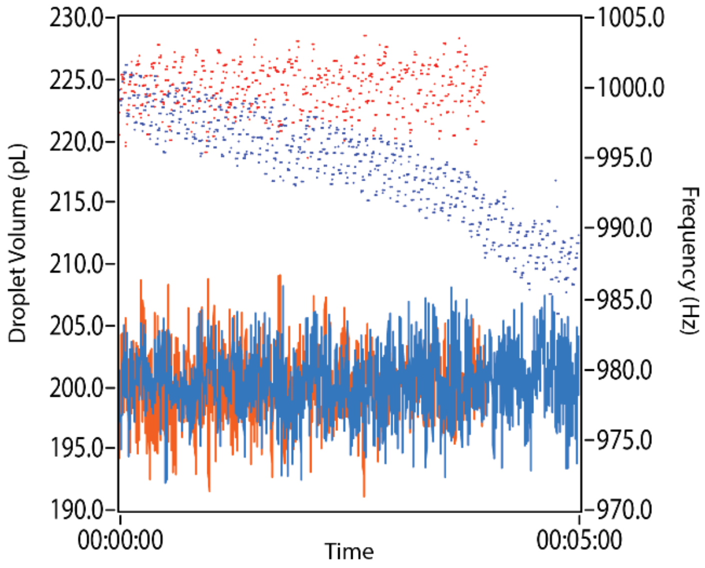

Figure S4 Droplet volumes (points) and frequencies (lines) of droplet creation both with (red) and without (blue) frequency feedback turned on. The droplet volume was set at 200 pL and the frequency of droplet generation starting off at 1 kHz. In both cases the droplet volume remained consistent but the frequency was found to drift if the frequency feedback was not present.

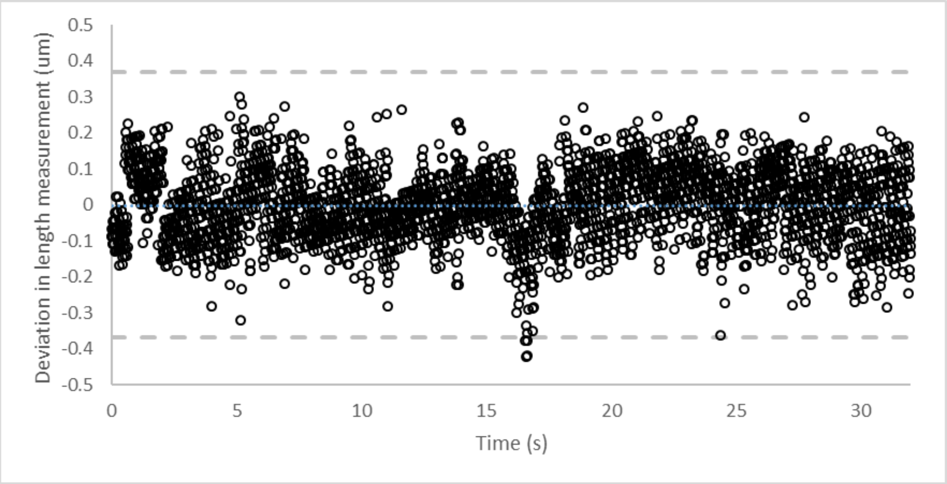

Figure S5 Measurement of the error in length estimation. A stationary droplet was imaged >3,600 times as the stage and camera oscillated and the length measured and volume calculated, grey lines represent the width of one pixel. The use of quadratic interpolation, gives sub-pixel accuracy of the length measurement, with a standard deviation of <100 nm.
